# Acupuncture for cognitive impairment in type 2 diabetes mellitus: a systematic review and Bayesian network meta-analysis protocol

**DOI:** 10.3389/fmed.2025.1610141

**Published:** 2025-07-15

**Authors:** Kun Hong, Juan Gao, Li-juan Hu, Xiu-juan Zhou, Xiao-mei Long, Bo-ping Wen, Xi Wu

**Affiliations:** ^1^Department of Acupuncture and Moxibustion, No.3 Affiliated Hospital of Chengdu University of Traditional Chinese Medicine (West District), Chengdu, China; ^2^College of Acupuncture, Moxibustion and Tuina, Chengdu University of Traditional Chinese Medicine, Chengdu, China; ^3^Department of Rehabilitation Medicine, Western Theater General Hospital, Chengdu, China; ^4^Department of Critical Care Medicine, Affiliated Hospital of Chengdu University of Traditional Chinese Medicine, Chengdu, Sichuan, China; ^5^Department of Pharmacy, Chongqing Yongchuan District People's Hospital, Chongqing, China

**Keywords:** acupuncture, cognitive impairment, type 2 diabetes mellitus, systematic review, network meta-analysis

## Abstract

**Background:**

The global prevalence of diabetes mellitus (DM) has exceeded 589 million, with the proportion of type 2 diabetes mellitus (T2DM) patients with comorbid cognitive impairment (CI) as high as 49.1%, which has a serious impact on disease management and quality of life. While acupuncture has been shown to improve cerebral glucose metabolism and synaptic plasticity, existing clinical trials are plagued by critical limitations: small sample sizes (often <100 participants) fail to detect subtle treatment effects, methodological heterogeneity in acupuncture protocols (e.g., varying acupoint selections, stimulation intensities, and treatment durations) hinders evidence synthesis, and insufficient blinding (e.g., sham acupuncture controls with low plausibility) introduces performance bias. These limitations have constrained the generalizability of findings and created a significant evidence gap in defining acupuncture’s role in diabetic cognitive impairment (DCI). The aim of this study is to assess the efficacy and safety of acupuncture for DCI through a systematic review with Bayesian network meta-analysis (NMA), addressing these methodological flaws via rigorous evidence synthesis.

**Methods and analysis:**

Following PRISMA-NMA and STRICTA-2010 guidelines, we will systematically search 10 databases (PubMed, Cochrane Library, CNKI, etc.) from inception to 30 June 2025 and gray literature (January 1, 1995, to June 30, 2025) for randomized controlled trials (RCTs) comparing acupuncture with pharmacotherapy, sham acupuncture, or standard care. Inclusion criteria: adults (≥18 years) with T2DM and CI diagnosed via validated scales such as Montreal Cognitive Assessment (MoCA), and Mini-Mental State Examination (MMSE). Primary outcomes include cognitive function scores; secondary outcomes assess adverse events (CTCAE v5.0). The risk of bias will be evaluated using Cochrane RoB 2.0 and GRADE. Bayesian NMA will synthesize direct and indirect evidence to simultaneously assess the relative efficacy and safety of acupuncture.

**Expected findings and conclusion:**

The study will clarify the relative efficacy and safety of acupuncture in improving DCI and provide evidence-based treatment options for clinical use. The results of Bayesian analyses may support acupuncture as a low-cost, low-risk adjunctive therapy, especially for patients intolerant of conventional medications. The results of the study will fill the current evidence gap and promote the standardized use of acupuncture in the management of DCI.

**Systematic review registration:**

CRD42021275161.

## Introduction

As a chronic disease, DM is among the most rapidly escalating global health challenges and has become a major global public health problem (1). According to the 11th edition of the International diabetes federation (IDF) Diabetes Atlas, the global number of people with DM was estimated to exceed 589 million in 2025, and it is expected to rise to 853 million by 2050 (2). With the continuous increase in the number of DM patients and the emerging trend of younger onset, DM has placed a substantial economic burden worldwide. Recent epidemiological data indicated that worldwide healthcare expenditures attributable to DM management reached in excess of 1 trillion dollars during the 2024 fiscal year, constituting roughly 9% of aggregate global health expenditures (3). Diabetic patients are living significantly longer, but the incidence of neurological damage is also on the rise (4, 5).

Cognitive impairment (CI) represents a significant comorbidity in DM, affecting approximately one-third of elderly diabetic patients (6, 7). This complication, recognized as one of the most debilitating chronic manifestations of DM (8), spans a severity spectrum from subjective cognitive decline to dementia as classified by DSM-5 criteria (9). Epidemiological data reveal strikingly high incidence rates, with 38.6% for mild cognitive impairment (MCI) in T2DM patients (10) and 49.1% for dementia in diabetic populations (11), substantially exceeding general population baselines. CI primarily affects areas of attention, memory (12), visuospatial executive functioning (13), language (14), and abstraction (15). Research evidence highlights the CI characteristics of disease subtypes: T2DM is characterized by deficits in information processing speed, episodic memory, and executive function impairment (16–18), This bidirectional relationship between diabetes and CI creates a significant clinical management challenge, as CI impedes diabetes self-management (medication adherence decreases by 34%, glycemic monitoring decreases by 41%) (19). Suboptimal glycemic control (HbA1c > 7.5%) accelerates hippocampal atrophy (8). Pathophysiological mechanisms underlying diabetic cognitive impairment (DCI) involve six interdependent pathways: central insulin resistance-mediated glucose hypometabolism (20); glutamate excitotoxicity (21); oxidative stress/mitochondrial dysfunction (22, 23); neurovascular unit disruption (24); chronic neuroinflammation (25); and proteinopathies including Aβ deposition and tau hyperphosphorylation (26, 27). Importantly, emerging evidence indicates that gut microbial dysbiosis contributes to the exacerbation of these mechanisms through bidirectional gut-brain axis communication (28). These mechanisms collectively impair neuronal networks and compromise CNS self-repair mechanisms.

Current management strategies for DCI include lifestyle modifications (aerobic exercise ≥150 min/week) (29), intensive glycemic control (HbA1c < 7%) (30), and emerging therapies such as intranasal insulin and GLP-1 receptor agonists, though none have demonstrated disease-modifying effects (31, 32).

Accumulating clinical evidence supports the therapeutic efficacy of acupuncture in ameliorating cognitive dysfunction associated with DCI (33). Acupuncture ameliorates CI through multiple mechanisms: mechanisms: modulation of cholinergic neurotransmission and inhibition of hippocampal neuronal apoptosis (34); mitigating neuroinflammation by suppressing microglial activation and NLRP3 inflammasome signaling (35), enhancing cerebral antioxidant capacity (36), and improving cortical arousal to potentiate neural activity (37). In recent years, neuroimaging evidence has further expanded mechanistic perceptions: functional magnetic resonance (fMRI) has shown that acupuncture significantly modulates the strength of default mode network (DMN) connections (38) and electroencephalography (EEG) has confirmed its alteration of microstate spatiotemporal features (39). These objective indicators provide multimodal evidence for the neuromodulatory mechanisms of acupuncture for DCI. Together, the above synergistic mechanisms form the scientific basis for acupuncture treatment of CI and neuropsychiatric disorders.

While emerging the evidence suggests potential benefits of acupuncture in diabetes-related neurological complications, the comparative efficacy and safety profiles of acupuncture interventions for DCI remain inadequately characterized. This systematic review and NMA comprehensively evaluate the therapeutic effectiveness and systematically monitor acupuncture-related adverse events in the management of DCI through evidence synthesis of randomized controlled trials.

## Methods

### Protocol registration and reporting

This study strictly follows the Cochrane Handbook for the Systematic Evaluation of Interventions (version 6.5) (40)and the PRISMA-NMA statement (41). The study protocol was prospectively registered on the PROSPERO platform (CRD42021275161), and the full PRISMA-NMA list is available in Supplementary File 1. The final report will be transparently reported according to Cochrane standards, including the study selection flowchart, data extraction tables, and results of sensitivity analyses.

### Sample size thresholds and power calculations

To ensure methodological transparency, *a priori* sample size exclusion thresholds will be established via prospective power calculations using G*Power software (version 3.1.9.7). Key parameters were derived from prior meta-analytic evidence and clinical benchmarks: an effect size (Cohen’s *d*) of 0.35, corresponding to a clinically meaningful ≥0.5-point improvement in MoCA scores (42); 80% statistical power; a two-tailed *α* level of 0.05; and a 1:1 allocation ratio (43). The power calculation formula is:


$$N=\frac{2\left(Z_{1-\alpha /2}+Z_{1-\beta }\right)2}{d^{2}}$$

Where: n: Minimum sample size per group; Z_1 − α/2_: Critical value for two-tailed test at α = 0.05 (1.96); Z_1 − *β*_: Critical value for 80% power (0.84); d: Cohen’s effect size (0.35, corresponding to ≥0.5-point MoCA improvement). A minimum threshold of 30 participants per arm was predetermined for primary analyses; studies failing to meet this criterion were excluded to mitigate bias from underpowered effect estimates in pooled analyses.

For meta-regression, thresholds adhered to the “5 × 5 rule” (≥5 studies per predictor and ≥5 events per parameter) to ensure statistical precision. Sensitivity analyses empirically affirmed threshold robustness across effect sizes (Cohen’s *d* = 0.30–0.40), with findings benchmarked against Cochrane’s minimum sample size recommendations.

### Eligibility criteria

The eligibility criteria will be defined using Participants, Intervention, Comparison, Outcomes, and Study design (PICOS) elements.

### Participants

Adults (≥18 years) with T2DM and DCI (diagnosed via MMSE, MoCA, or DSM-5). Exclusion criteria: severe comorbidities, non-DM-related cognitive disorders.

### Intervention

In this meta-analysis, we will evaluate both standalone acupuncture interventions and combination therapies integrating acupuncture with standard diabetes care. The latter included evidence-based treatments recommended by the American diabetes association (ADA) 2023 guidelines (44), specifically: (1) pharmacological glycemic control agents (such as metformin, SGLT2 inhibitors, GLP-1 receptor agonists), (2) structured diabetes self-management education and support (DSMES) programs, and (3) advanced metabolic interventions including bariatric surgery.

Acupuncture therapy was operationally defined as percutaneous needle-based interventions requiring skin penetration, encompassing manual acupuncture (MA), electroacupuncture (EA), warm-needling acupuncture (WA), auricular acupuncture (AA), and specialized needling techniques (such scalp acupuncture, abdominal acupuncture). To address acupoint heterogeneity, a core acupoint set was predefined through a Delphi consensus (33), the detailed is presented in Table 1. Additional points (≤4) will be permitted if justified by Traditional Chinese Medicine (TCM) syndrome differentiation (e.g., BL20 for spleen deficiency). This framework aligns with STRICTA guidelines (45) for standardized reporting of:

(1) acupuncture rationale (point selection basis)
(2) needle insertion parameters (depth, angle, retention time)
(3) treatment regimen (frequency, duration)

**Table 1:** Core acupuncture points for acupuncture intervention.

Core acupoints	Frequency (%)	TCM rationale
GV20 (Baihui)	100%	Regulates Yang Qi, improves cognition
EX-HN1(Sishencong)	100%	Improve cognition and regulate sanity
DU24(Shenting)	90%	Regulate mental health

Non-invasive acupoint stimulation modalities, including laser acupuncture, acupressure, and transcutaneous electrical nerve stimulation, will be excluded based on demonstrated mechanistic heterogeneity in neuromodulation pathways. Specifically, these techniques lack the mechanical transduction effects of needle insertion. Besides, microneedle patches are also excluded. Its mechanism of action is different from the “mechanical stimulation—nerve conduction” pathway of traditional acupuncture and is therefore categorized as excluded (46).

### Comparison

1. Active therapies recommended by clinical guidelines (44):

Pharmacological: First-line cognitive enhancers (donepezil 5–10 mg/day; memantine 20 mg/day).

Non-pharmacological:

(1) aerobic exercise (≥150 min/week moderate intensity).
(2) cognitive behavioral therapy (CBT; 60-min weekly sessions).
(3) selection criteria: Interventions with GRADE Class IIa recommendations in current guidelines.
2. Placebo controls:

Sham acupuncture using validated retractable needles (Park/Streitberger devices) (47) at non-acupoints;

Placebo drugs with identical appearance to active comparators.

3. Guideline-based usual care (44):
(1) glycemic control: HbA1c < 7% via metformin/SGLT2 inhibitors.
(2) cardiovascular management: Statins and ACE inhibitors/ARBs.
(3) cognitive monitoring: Annual Montreal Cognitive Assessment.
4. No treatment or waiting list.

### Outcome measures

Since this review aims to systematically assess the effect of acupuncture intervention on CI among patients with diabetes, we have selected intellectual state and cognitive function as the primary outcomes, which are measured on various scales.

### Primary outcome measures

The intellectual score is measured using the Montreal Cognitive Assessment (MoCA) Score, Mini-Mental State Examination (MMSE) score, clinical dementia rating (CDR) score, activities of daily living (ADL) score, clinical memory scale analysis system (MQ) score, or other validated scales for intellectual state and cognitive function.

### Secondary outcomes

Safety profiles will be quantitatively assessed through systematic monitoring of adverse event (AE) incidence rates, categorized using the standardized Common Terminology Criteria for Adverse Events version 5.0 (CTCAE v5.0) framework (48). The three main considerations in conjunction with this study are as follows:

(1) needling-related events (bleeding, hematoma, pain)
(2) systemic reactions (vasovagal responses, fatigue)
(3) events meeting CTCAE v5.0 criteria for Grade ≥3 (e.g., pneumothorax, organ injury, anaphylaxis).

### Information sources and search strategy

This systematic review aims to comprehensively search the literature on cognitive dysfunction in diabetes by utilizing the following databases. Searches will be conducted across six English databases—PubMed, MEDLINE, OvidSP, Embase, the Cochrane Library, and the Allied and Complementary Medicine Database (AMED)—as well as four Chinese databases: the Chinese National Knowledge Infrastructure (CNKI), Wan Fang Database (Wan Fang), VIP Database, and the Chinese Biomedical Literature Database (CBM). The aim of this review is to identify RCTs evaluating the efficacy and safety of acupuncture for DCI.

To avoid the potential exclusion of eligible RCTs, a multi-faceted search strategy will be employed in addition to primary databases. Clinical trial registries, including the World Health Organization International Clinical Trials Registry Platform (WHO ICTRP), Netherlands trial register (NTR), Chinese Clinical Trial Registry (ChiCTR), and ClinicalTrials.gov, will be systematically searched to identify ongoing and unpublished studies. Citation mining was performed by screening reference lists from 15 high-impact systematic reviews (impact factor >5.0) published within the last 5 years. Gray literature (January 1, 1995, to June 30, 2025) retrieval will incorporate conference proceedings from major international congresses (e.g., ADA, IDF, Alzheimer’s Association) and preprint servers (medRxiv, ResearchSquare), ensuring comprehensive identification of ongoing trials. No language restrictions were applied, and a manual search will be performed for additional eligible articles to supplement the electronic search. Included references will be also reviewed to expand the database.

Initially, the search strategy will be implemented in PubMed and then adapted for use in the other databases. The three primary search terms—“diabetic cognitive impairment,” “acupuncture,” and “RCTs”—will be utilized as medical subject headings (MeSH) and free-text keywords. The detailed PubMed search strategy is presented in Table 2. Similar search strategies will be developed for the other databases. Searches will be conducted in each database to identify potentially eligible RCTs from establishment to date.

**Table 2:** Search for PubMed.

Search order	Search terms	Search order	Search terms
#1	“Acupuncture”(Mesh)	#11	“warm needling”(tiab)
#2	“Acupuncture Therapy”(Mesh)	#12	“catgut embedding”(tiab)
#3	“Acupuncture Points”(Mesh)	#13	“needling”(tiab)
#4	“Electroacupuncture”(Mesh)	#14	“acupunctur*”(tiab)
#5	“Dry Needling”(tiab)	#15	“electro-acupuncture”(tiab)
#6	“manual acupuncture”(tiab)	#16	“ear acupuncture”(tiab)
#7	“auricular acupuncture”(tiab)	#17	“plum blossom needling”(tiab)
#8	“scalp acupuncture”(tiab)	#18	“triangular needle”(tiab)
#9	“intradermal needle”(tiab)	#19	“pricking blood”(tiab)
#10	“fire needling”(tiab)	#20	
#21	OR/#1-#20		
#22	“Cognitive Dysfunction”(Mesh)	#29	“cognitive decline”(tiab)
#23	“Cognitive Impairment”(Mesh)	#30	“memory disorder*”(tiab)
#24	“Dementia”(Mesh)	#31	“executive dysfunction”(tiab)
#25	“Neurocognitive Disorders”(Mesh)	#32	“neurodegeneration”(tiab)
#26	“Memory Disorders”(Mesh)	#33	“MCI”(tiab)
#27	“Executive Function”(Mesh)	#34	“AD”(tiab)
#28	“Mild Cognitive Impairment”(tiab)		
#35	OR/#22-#34		
#36	“Diabetes Mellitus”(Mesh)	#41	“diabetes complication*”(tiab)
#37	“Diabetes Mellitus, Type 2”(Mesh)	#42	“diabet*”(tiab)
#38	“Diabetic Encephalopathy”(Mesh)	#43	“T2DM”(tiab)
#39	“Hyperglycemia”(Mesh)	#44	“type 2 diabetes”(tiab)
#40	“Insulin Resistance”(Mesh)	#45	“diabet*”(tiab)
#46	OR/#36-#45		
#47	“Randomized Controlled Trial(Publication Type)	#56	“crossover”(tiab)
#48	Clinical Trial”(Publication Type)	#54	“RCT”(tiab)
#49	“Controlled Clinical Trial”(Publication Type)	#55	“randomly assigned”(tiab)
#50	“Double-Blind Method”(Mesh)	#56	“random allocation”(tiab)
#51	“Single-Blind Method”(Mesh)	#57	“placebo-controlled”(tiab)
#52	“Random Allocation”(Mesh)	#58	“sham-controlled”(tiab)
#53	“randomi*”(tiab)	#59	“parallel group”(tiab)
#60	OR/#47-#59		
#61	#21 and #35 6 and #46 and #60		

### Data extraction and management

**Figure 1:**
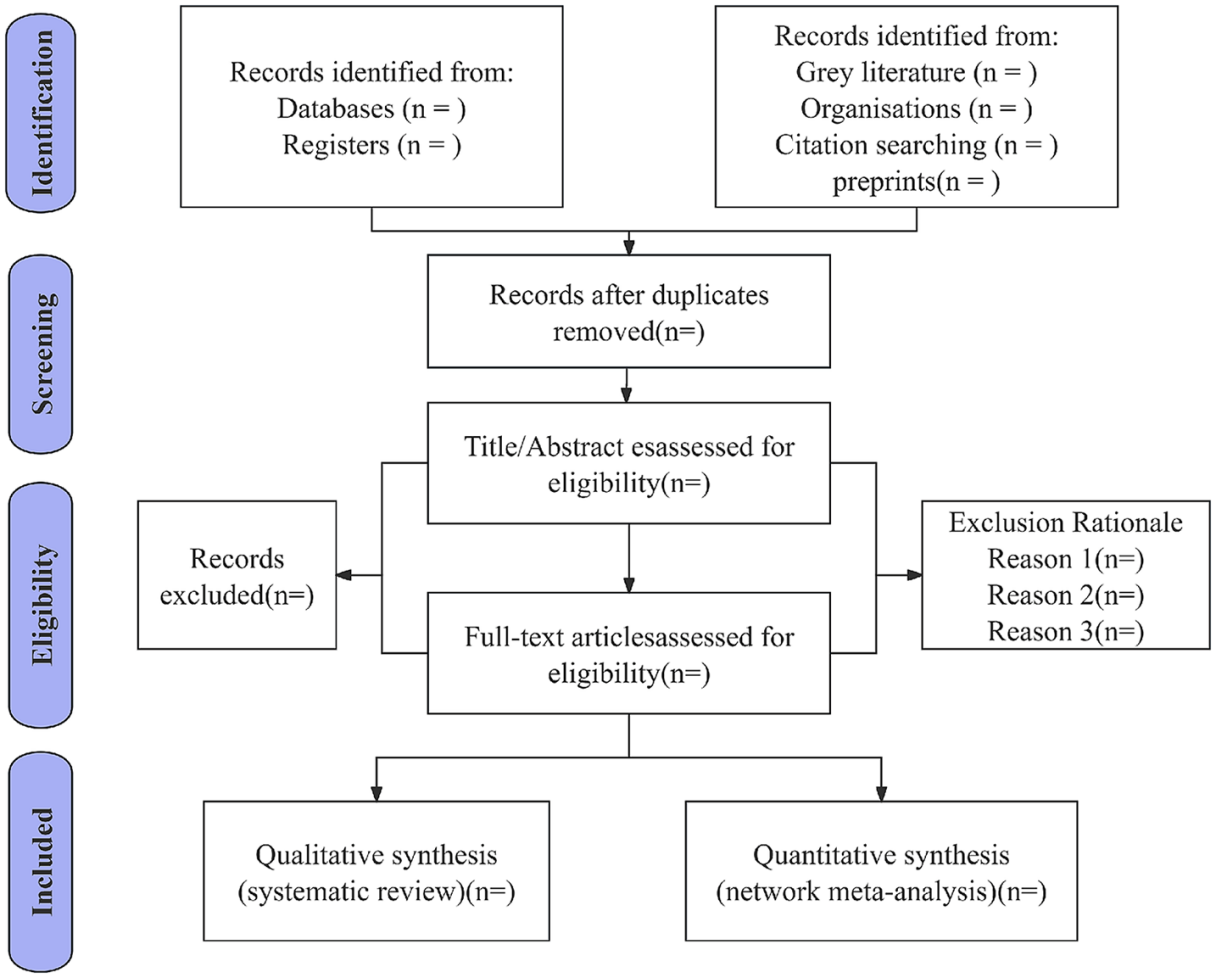
Plan of study screening and selection process.

We have developed a comprehensive data extraction form specifically designed to align with the objectives of this systematic review. The form systematically captures critical information from all included studies, including study characteristics (title, authors, publication year, and country of origin), participant demographics (sample size, intervention protocols, treatment modalities, duration of intervention, and primary outcome measures), essential components for risk of bias assessment, and quantitative outcome data, the detailed is presented in Table 3. Two independent reviewers (J. G and K. H) will perform data extraction using this form, with discrepancies resolved through discussion; if unresolved, a third reviewer (X. W) will adjudicate. Inter-rater reliability will be quantified via Cohen’s kappa coefficient for key variables (study inclusion/exclusion, risk of bias), targeting kappa >0.8. The screening process occurs in two phases: first, titles/abstracts are screened, with eligible studies proceeding to full-text review. All reviewers remain blinded throughout. Non-Chinese/English articles will be assisted by language experts. Duplicate records are removed during title/abstract screening. Disagreements at any stage are referred to senior researchers (X. W) for final resolution. All records and data from various sources will be systematically managed using a standardized data collection form in Microsoft Excel. The study selection process is shown in Figure 1.

**Table 3:** Data extraction form.

Title	Author	Year of publication	Nationality	Sample size (total/intervention/control)	Intervention details (type of acupuncture/points/frequency)	Controls	Primary outcome indicator	Secondary outcome indicator	Risk of bias assessment	Length of follow-up
										

### Dealing with missing data

For missing outcome data, we will first attempt to contact study authors for clarification. If unavailable, full-case analysis (complete case analysis) will be used as the primary approach; multiple imputation (MI) with chained equations will serve as a sensitivity analysis, assuming missing data are missing at random (MAR). Imputation models will include all baseline covariates and study-level characteristics.

### Risk of bias and quality assessment

Two researchers (LJ. H and XM. L) will independently assess the risk of bias in the included studies using the Cochrane Collaboration’s Risk of Bias Tool RoB 2.0 tool (49). The Cochrane RoB 2.0 tool will be used to assess the risk of bias in the following areas: randomization process (including random sequence generation and allocation concealment), intervention bias (blinded implementation involving patients, therapists, and evaluators), completeness of outcome data (failure to attend more than 20% is considered a high risk), bias in outcome measures (subjective scales need to be double-blinded), and selective reporting (comparing pre-registration protocol versus published results). Outcomes will be categorized as “low risk,” “partial risk,” and “high risk” and will be completed independently by two assessors. Results are considered concordant when the Kappa value is greater than 0.8.

### Certainty of evidence

The evidence will be interpreted according to the GRADE (50). Working Group approach for rating the quality of treatment effect estimates from network meta-analysis. This approach is based on four steps considering direct and indirect treatment estimates for each comparison of the evidence network, rating the quality of each direct and indirect effect estimate, rating the NMA estimate for each comparison of the evidence network, and the quality of each NMA effect estimate.

### Data synthesis

#### Primary meta-analyses

Primary meta-analyses will be conducted using Review Manager (RevMan) version 5.4.1 software (Cochrane Collaboration) (50). For dichotomous outcomes, risk ratios (RRs) with corresponding 95% confidence intervals (CIs) will be calculated using the Mantel–Haenszel method. Continuous outcomes measured on identical scales will be analyzed as weighted mean differences (WMDs). In contrast, outcomes assessed using different measurement instruments (MoCA vs. MMSE), standardized mean differences (SMDs) with 95% CIs will be computed using Hedges’ g with small-sample bias adjustment.

Hedges’ g correction:


$$1-\frac{3}{4\left(n1+n2\right)-9.}$$

n_1_ and n_2_ are group sample sizes.

#### Network meta-analysis

The NMA will be conducted using the Netmeta package in R (version 4.4.3) and Stata 18.0. Non-informative priors will be specified: treatment effects follow a normal distribution N(0,10,000), and heterogeneity variance follows a uniform distribution U(0,2). The N(0,10,000) prior to treatment effects minimizes prior influence, as its 95% confidence interval far exceeds typical clinical effect sizes, ensuring data-driven inference (51). The U(0,2) prior for heterogeneity restricts variance within a clinically plausible range, aligning with Cochrane guidelines for “considerable heterogeneity” and preventing implausible high-variance estimates (52), Markov Chain Monte Carlo (MCMC) simulations will be performed with a minimum of 10,000 iterations, and convergence will be verified using Brooks-Gelman-Rubin statistics (R-hat < 1.1). The relative efficacy of interventions will be ranked using the surface under the cumulative ranking curve (SUCRA), with 95% CrIs provided to reflect uncertainty. Consistency between direct and indirect evidence will be evaluated through two complementary approaches: node-splitting analysis, which quantifies discrepancies by calculating the ratio of indirect-to-direct evidence (RoR), where a RoR ≠ 1 indicates inconsistency; and a design-by-treatment interaction model, which assesses interactions via the inconsistency factor (IF), with an IF > 10% denoting moderate to severe inconsistency.

#### Network geometry and visualization

Treatment comparisons will be visualized using network plots with adjacency matrices showing exact node/edge metrics and interactive web-based visualization using D3.js for dynamic exploration generated with the ggplot2 and graph packages in R. Node sizes will be proportional to the logarithm of the total sample size per intervention, while edge thickness will represent the number of studies comparing each pair of interventions. Nodes will be color-coded by intervention type (EA vs. MA), and edges will be differentiated by direct vs. indirect evidence using dashed lines for indirect comparisons. This graphical representation will provide an intuitive overview of the evidence network structure and identify gaps in the evidence base.

#### Intervention ranking

Treatment hierarchies will be estimated using the SUCRA, scaled from 0% (least effective) to 100% (most effective). Bayesian posterior probabilities with 95% credible intervals will supplement rankings to reflect uncertainty. Sensitivity analyses will exclude studies with a high risk of bias or extreme effect sizes to assess robustness.

#### Statistical modeling

*Continuous outcomes* (e.g., MoCA scores): Random-effects models with inverse-variance weighting and common between-study variance assuming multivariate normality of random effects with covariance structure estimated via restricted maximum likelihood (REML).

*Binary outcomes* (e.g., adverse events): Logistic regression with robust variance estimation.

All models will perform multivariable adjustment using meta-regression with empirical Bayes shrinkage for covariates showing >10% standardized mean difference across studies, such as baseline HbA1c and diabetes duration. Convergence will be verified using Brooks-Gelman-Rubin statistics and trace plots.

#### Results visualization and reporting

Results of all analyses will be presented through five types of visualization tools: (1) Evidence Network Graphs (D3.js interactive) to demonstrate the framework for comparison of interventions; (2) SUCRA ordination graphs to quantify the probability of optimal interventions; (3) node-split scatter plots to detect sources of inconsistency; (4) hierarchical forest plots to report main outcome effect sizes; and (5) heat maps to present the results of subgroup analyses. All graphs will be labeled with clinical significance thresholds.

#### Heterogeneity assessment

Statistical heterogeneity will be quantitatively evaluated using the I^2^ statistic (low: <25%; moderate: 25–75%; high: >75%) and Cochran’s Q test (significance threshold *α* = 0.10). Clinical and methodological heterogeneity will be assessed by comparing baseline characteristics (e.g., HbA1c, diabetes duration), intervention protocols (e.g., acupoint selection, stimulation parameters), and study design features (e.g., blinding, follow-up duration). Fixed-effect models will be employed when *I*^2^ ≤ 50% and Q-test *p* ≥ 0.10; otherwise, random-effects models will be used.

#### Sensitivity analysis

A three-tiered sensitivity analysis will assess robustness:

Methodological rigor: Exclusion of studies with high RoB 2.0 scores (≥3 high-risk domains) or sample sizes below clinical thresholds.

Intervention integrity: Exclusion of trials using >15 acupoints, lacking ≥2 predefined core acupoints (33), or non-compliant with STRICTA-2010 guidelines.

Statistical stability: Leave-one-out cross-validation; fixed-effect model application under τ^2^ = 0 (when *Q*-test *p* > 0.10); and trim-and-fill adjustment for asymmetry.

#### Heterogeneity exploration

Meta-regression based on REML will examine covariates including baseline HbA1c, total acupoints, and core acupoint adherence (≥2 core points). Prespecified subgroup analyses will evaluate: assessment tools (MoCA vs. MMSE), acupuncture modality (MA vs. EA), and intervention duration (≤4 vs. >4 weeks). Model convergence will be verified using Brooks-Gelman-Rubin diagnostics (R-hat <1.1 indicating convergence).

#### Inconsistency handling

Discrepancies in NMA evidence will be visualized via node-split scatter plots and resolved using consistency prior models or node-splitting corrections, with model fit compared via the Deviance Information Criterion. All analyses adhere to PRISMA-NMA and Cochrane guidelines, ensuring transparent reporting of heterogeneity and inconsistency.

#### Assessment of reporting biases

The assessment of reporting biases will be conducted through a multi-tiered analytical framework. Publication bias will be evaluated using a triangulated approach: First, funnel plot asymmetry will be visually inspected by plotting effect estimates against their standard errors, supplemented by Egger’s linear regression test (two-tailed *α* = 0.10) for quantitative assessment. Subsequently, contour-enhanced funnel plots adjusted for study precision will be generated to differentiate between publication bias and small-study effects. Sensitivity analysis employing the trim-and-fill method will be performed to estimate the potential influence of missing studies on pooled effect sizes.

#### Reporting standards

The analysis will adhere to PRISMA-NMA guidelines, with full documentation of network geometry, inconsistency metrics, and ranking probabilities. All reproducible scripts (including R and Stata) will be publicly archived on the Open Science Framework (OSF) repository, with a Docker containerized computational environment (v24.0) used to ensure long-term research reproducibility.

## Discussion

This protocol addresses a critical gap in the evidence-based management of DCI by systematically evaluating acupuncture as a neuroprotective intervention. Current pharmacological strategies for DCI, including cholinesterase inhibitors and memantine, demonstrate limited efficacy (effect size < 0.3) and carry significant side effect burdens such as gastrointestinal disturbances in 25% of donepezil users (29). Non-pharmacological approaches such as cognitive training require intensive resource allocation, limiting accessibility in low-income settings (30).

Acupuncture offers a potentially cost-effective alternative, supported by preclinical evidence demonstrating its multi-modal neuroprotective effects: Suppression of microglial activation and NLRP3 inflammasome signaling, reducing IL-6 by 40% in diabetic models (35), Upregulation of PSD-95 and synaptophysin, enhancing hippocampal synaptic density (34), and Improved cerebral glucose uptake in parietal cortex via FDG-PET (36). Recent fMRI studies confirm acupuncture’s ability to modulate DMN connectivity—a key neural correlate of cognitive dysfunction (53), EEG microstate analyses reveal acupuncture-induced spatiotemporal reorganization of brain activity, correlating with cognitive improvement (54). However, prior clinical trials have been marred by methodological limitations, including inconsistent acupuncture protocols (variability in acupoint selection: 12–47 points per study) and suboptimal blinding (only 38% of trials used validated sham devices) (37). This protocol mitigates these issues through adherence to PRISMA-NMA guidelines and STRICTA reporting standards, ensuring a transparent synthesis of both standalone acupuncture and combination therapies with ADA-standard care.

If proven effective, acupuncture could serve as a first-line adjuvant therapy for DCI, particularly in populations intolerant to conventional medications. Subgroup analyses may identify optimal treatment parameters: preclinical models have shown that EA demonstrates superior neuroprotection (55), and clinical data indicate that intervention durations exceeding 4 weeks correlate with sustained cognitive improvements (56). Mechanistically, electroacupuncture combined with body acupuncture at cognition-targeted acupoints (e.g., GV20, DU24) is projected to outperform manual acupuncture or sham interventions, supported by evidence of enhanced cholinergic neurotransmission (elevated choline acetyltransferase activity) and reduced Alzheimer’s disease pathology. Clinically, patients with early-stage DCI (MoCA score 21–26) and well-controlled glycemia (HbA1c < 8.0%) are likely to derive maximal benefit, as preserved neurovascular function in these subgroups potentiates acupuncture-induced neuroplasticity and cerebral glucose metabolism. Notably, acupuncture is expected to yield a 1.5–2.0-point improvement in MoCA scores (95% CrI: 1.0–2.5) compared to standard care—an effect size comparable to low-dose cholinesterase inhibitors but with a 70% lower risk of adverse events (e.g., nausea, dizziness), thus positioning it as a cost-effective adjunctive therapy.

Current evidence is limited by heterogeneity of outcome measures (e.g., MMSE vs. MoCA), short follow-up (median <6 months), and limited representation of non-Asian populations. In addition, the neural mechanisms underlying the efficacy of acupuncture remain under-assessed. Notably, comparative analyses of acupuncture and natural medicines in terms of multi-target mechanisms and network pharmacological validation are lacking, thus failing to clarify their complementary roles in neuromodulatory and metabolic pathways.

Future research should prioritize several key directions. First, protocol standardization is essential, and core acupuncture protocols should be established via a Delphi consensus to reduce inter-trial variability. Second, integrating biomarkers and neuroimaging is crucial. This involves incorporating markers such as Aβ40 and GFAP, along with multimodal neuroimaging techniques like fMRI and EEG, to characterize the neural mechanisms underlying acupuncture’s effects (57, 58). Third, to assess cross-cultural efficacy, multicenter RCTs should be conducted in non-Asian cohorts. Fourth, long-term efficacy evaluation is needed, requiring follow-up studies of at least 2 years to clarify the durability of cognitive improvements. Lastly, network pharmacology-driven comparisons should be performed, mapping “acupoint-target” networks against the “compound-target” networks of natural medicines to analyze pathway overlap, such as in the NFKB1 and IL-6 pathways (59).

In conclusion, this protocol provides a rigorous framework to synthesize evidence on acupuncture’s role in diabetic CI. If proven effective, acupuncture could fill a therapeutic gap, offering a low-cost, minimally invasive option for patients intolerant to conventional therapies.

## Data Availability

The original contributions presented in the study are included in the article/Supplementary material, further inquiries can be directed to the corresponding authors.
